# 

**Published:** 1895-11-30

**Authors:** 


### CONTENTS.

Restrictions in the Use of Unproved Remedies - - 141
Dr. Waldo and the Salvation Army - - - - - 142
Administration of Anæsthetics.—II. By Sir B. W. Richardson, M.D. - - - - - 143
Modern Sociology.—Climates of the Past - - - - - 144
Medical Progress and Hospital Clinics—
Sleep and its Disorders.—I. - - - - - - 147
Progress in Medicine.—Typhoid Fever - - - - 148
Progress in Surgery.—General Surgery - - - - 148
Progress in Otology - - - - - - - 151
New Appliances and Things Medical - - - - 152
Annotations.—Chemical Sterilization of Water—The Wholesale Poisoners of London—Salvation Army Shelters - - - - 145
Notes and News - - - - - - - - - 146
The Institutional Workshop—
Hospital Expenditure. — The Commissariat. VIII.—The Nurses' Table—London - - - - - 153
Practical Departments. III.—A New Lifting Seat and Carrying Chair - - - - - - - 155
Hospital Meetings.—Hospital Sunday Fund - - - - 156
Scraps and Gleanings - - - - - - - - 156
The Book World of Medicine and Science - - - 157

### EXTRA SUPPLEMENT.—THE NURSING MIRROR.

News from the Nursing World.—Christmas Decorations —Our Needlework Competition—A Vacant Matronship at Brighton—Loughton Convalescent Home—Empty Cupboards—Christmas Festivities—Combined Day and Night Duty—Maternity Charity, Plaistow—An Abuse and a Danger—Nurses at Glasgow—Queen's Nurses at Dundee—Easily Satisfied—New Nursing Sisterhoods—Short Items - - - - - - lxix

French Schools for Trained Nurses: Their Origin and Organisation - - - - - - - lxxii

Elementary Physiology for Nurses. By C. F. Marshall, late Surgical Registrar, Hospital for Sick Children, Great Ormond Street. XVII.—The Eye and the Sense of Sight - lxxi

Royal British Nurses Association - - - - - lxxi

The Princess Maud Marriage Present Fund - - lxxiii

Royal National Pension Fund for Nurses - - - lxxiii

Novelties for Nurses - - - - - - - - - lxxiv

Where to Go - - - - - - - - - - - lxxiv

Notes and Queries - - - - - - - - - lxxiv

